# Conservative Management of Invasive Cervical Resorption: A Case Report

**Published:** 2013-05

**Authors:** Fahad Umer, Samira Adnan, Farhan Raza Khan

**Affiliations:** 1 Senior Instructor, Operative Dentistry Program, Dental Section, Aga Khan University Hospital, Karachi, Pakistan; 2 Resident, Operative Dentistry Program, Dental Section, Aga Khan University Hospital, Karachi, Pakistan; 3 Assistant Professor, Operative Dentistry Program, Dental Section, Aga Khan University Hospital, Karachi, Pakistan

**Keywords:** Calcium Hydroxide; Root Resorption; Root-Canal Medicaments; Root Canal Therapy

## Abstract

Invasive cervical resorption is a condition that affects the root surface area below the epithelial attachment. Multiple treatment modalities are advocated, involving exposure of the invasive defect, removal of the granulation tissue and sealing with various restorative materials. This report demonstrates conservative treatment of a patient presenting with peri-apical periodontitis in upper right central and lateral incisors, along with Class II invasive resorption defect cervically on the mesial aspect of the central incisor, as a result of trauma. As the patient was not willing for any surgical intervention, only ortho-grade root canal treatment was carried out in both teeth, with Calcium hydroxide as intra-canal medicament. At three year follow-up, the patient remains asymptomatic demonstrating radiographic evidence of infilling of defect with bone-like tissue.

Within the limitations of this report, it was seen that this conservative method for halting the progression of invasive cervical resorption could be under taken in patients who are un-willing for surgical intervention or in whom surgery is contra-indicated.

## Introduction

Invasive cervical resorption (ICR) is a condition that affects the root surface area below the epithelial attachment. It is defined as “a localized resorptive process that commences on the surface of the root below the epithelial attachment and the coronal aspect of the supporting alveolar process, namely the zone of the connective tissue attachment” [1]. 

This terminology was preferred by Heithersay [2], as the condition is both invasive and aggressive, but other terms have also been used in literature to describe this form of external resorption [3] (Table 1). Patients presenting with this condition are usually asymptomatic unless there is super-imposition of pulpal or periodontal infection secondary to invasion of the lesion into the pulp. Clinically, it may present as a pink discoloration on the tooth’s cervical region as the highly vascular granular tissue becomes visible through the resorbed tooth structure. 

It is usually detected as a co-incidental radiographic finding during routine dental examination [2].

Although several etiological factors have been implicated, including dento-alveolar surgery and periodontal treatment, the most common predisposing factor triggering this type of resorption is considered to be orthodontics, followed by trauma and intra-coronal bleaching [4]. It is essential to differentiate ICR from internal resorption in order to reach the appropriate diagnosis and to offer a valid treatment plan to the patient [5].

 Clinical features, along with radiographical findings, are paramount in formulating a diagnosis. Peri-apical radiographs, using parallex technique, can be taken in order to follow the outline indicating the continuity of the pulp chamber. 

In internal resorption, the defect will remain centered on the root canal, while in ICR the lesion will seem to move with the Xray tube angulation.[6]. 

Alternatively, cone beam computerized tomography (CBCT) can be used, which improves the diagnostic yield when compared to conventional radiographs and may also help to accurately determine the extent, depth and dimension of the lesion [7]. 

Treatment of ICR, as advocated by Heithersay, involves reflection of flap to expose the defect, application of 90% aqueous solution of trichloracetic acid to the resorptive tissue, curettage, endodontic treatment where necessary, followed by restoration [2].

## CASE REPORT

**Fig 1:**
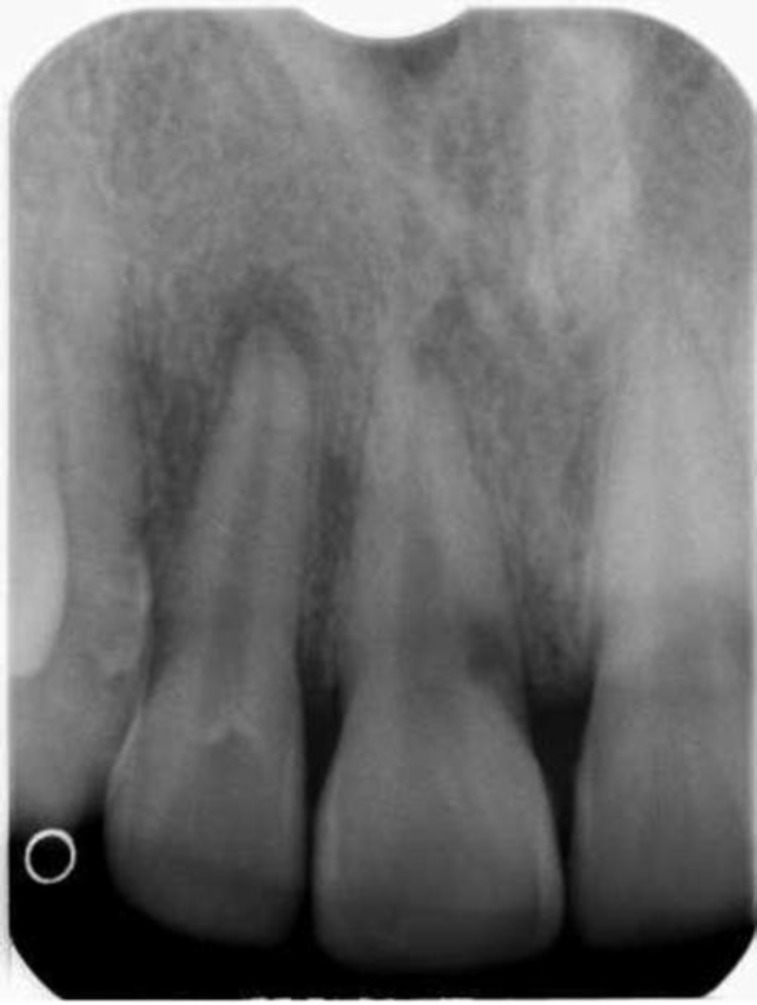
Pre-operative Radiograph

A 23-year-old South East Asian male presented for consultation at the endodontic department (under the care of Operative Dentistry Postgraduate Residency program) in 2008 with the chief complaint of discolored upper right central and lateral incisors. The patient’s medical history was non-contributory. The patient had a history of trauma to his upper teeth, due to falling 6-8 years before. Sensibility tests were performed on both teeth, which yielded negative response. Hence, tooth # 11 and #12 were considered non-vital as a result of trauma. On probing with a CPITN-C probe, the probing depth for both teeth was within normal limits. The peri-apical radiograph revealed peri-apical radiolucency associated with #11 and #12, and an irregular cervical radiolucent defect present on the mesial aspect of tooth #11, extending from the cement-enamel junction (Fig 1). Diagnosis of chronic periapical periodontitis secondary to pulpal necrosis was made for both tooth#11 and #12, with ICR in tooth #11.

The patient was informed of the diagnosis, the treatment plan along with the alternatives, and the p However, the patient did not consent to any surgical treatment. Therefore, it was decided to proceed with non-surgical root canal treatment only prognosis of the case was explained.

Non-surgical root canal therapy was initiated under local anesthesia 2% xylestesin 1:80,000 dilution (3M ESPE), (two cartridges of 1.8ml) given as infiltration in the vestibular sulcus.

**Table 1:** Terms Used to Describe Invasive Cervical Resorption

1. Odontoclastoma 2. Peripheral cervical resorption 3. Extra-canal invasive resorption 4. Supra-osseous extra-canal invasive resorption 5. Peripheral inflammatory root resorption 6. Sub-epithelial external root resorption

Lingual access opening was made in both teeth using round diamond bur #4 that was extended with a tapered safe-ended diamond bur (Mani dental diamond burs, China). The teeth were instrumented with ISO standard H files (stainless steel, 0.02 taper) and the working length was established with the help of both Apex locator (Root ZX II J.Morita, U.S.A) and a radiograph. After the working length was determined, the master apical file was established as #60 in tooth #12 and #70 in tooth #11. Recapitulation was done between each instrument usage, irrigating the canal with 2 ml of 4% sodium hypochlorite each time. Both canals were prepared using a traditional step back technique using files #70, 80 and 90 (1mm, 2mm and 3mm short of the working length, respectively) for tooth #12 and files #80, 90 and 100 (1mm, 2mm and 3mm short of the working length, respectively) for tooth #11.

Calcium hydroxide (Metapex, Biomed Co. Ltd, South Korea) was placed as inter-appointment dressing and the cavity was closed with Cavit (3M ESPE) temporary restoring material.

**Fig 2:**
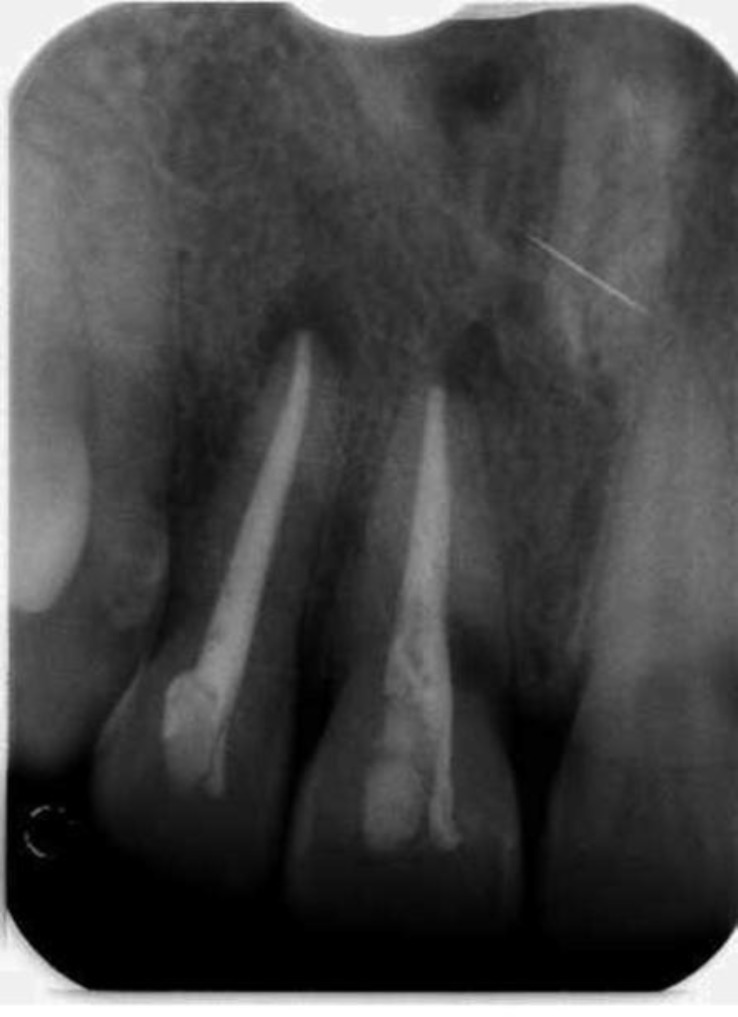
Immediate post-op Radiograph

The patient was recalled after fifteen days for the next appointment. On the subsequent visit, calcium hydroxide was removed with the help of ISO standard H files (stainless steel, 0.02 taper, #50, 55, 60, 70)) and after drying the canal with absorbent paper points, the canals of both teeth were obturated using ISO standard gutta percha points (Detrey, Dentsply) employing cold lateral condensation techniques, with Seal-apex (Kerr Co. Orange, CA) as root canal sealer. The access cavity was sealed using composite resin (Valux , 3M ESPE) (Fig 2).

Although both teeth remained discolored after endodontic treatment, intra-coronal bleaching was not selected as an option to improve the appearance of the teeth, as it is considered as one of the etiological factors initiating cervical resorption. 

The patient was informed of this possible adverse outcome and was offered other cosmetic treatments including direct or indirect composite veneers, porcelain veneers or porcelain jacket crowns, but he was not interested in such procedures involving any degree of tooth preparation.

**Fig 3:**
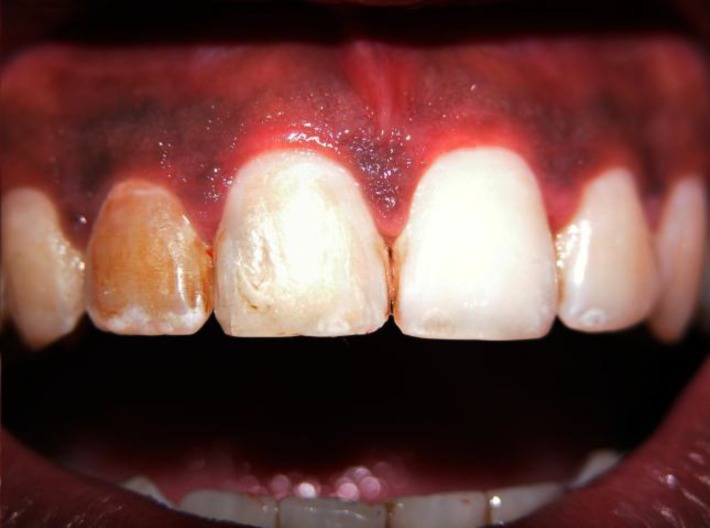
Post op Intra-oral photograph

**Fig 4:**
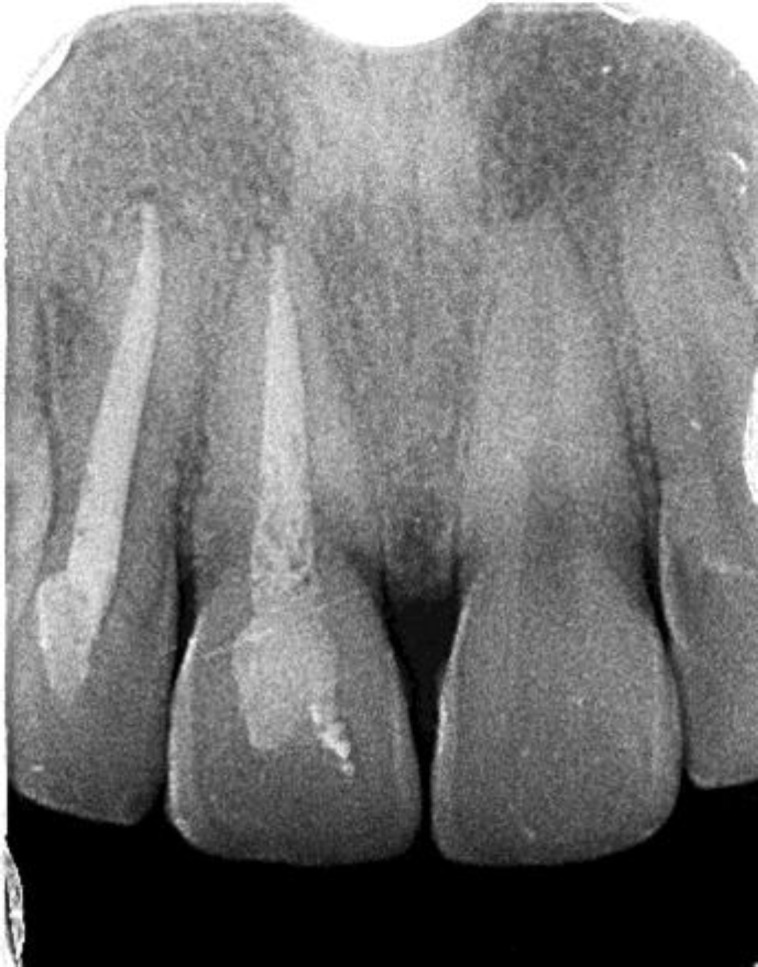
Radiograph at 3-year follow-up

Hence after discussion, the patient’s teeth were left discolored. At the three year follow-up, the tooth has remained completely asyptomatic and the intra-oral peri-apical radiograph showed complete healing of periapical radiolucency along with improved radiopacity of the cervical defect (Fig 3 and 4). 

## Discussion

In this treated case, invasive cervical resorption was most likely caused due to the trauma reported by the patient in his early childhood. As invasive cervical resorption is detected much later than the time of the initial trauma, the correlation between the two may not be realized immediately. 

According to a study conducted by Heithersay [8], 15.1% of the teeth undergoing trauma subsequently developed invasive cervical resorption. Most frequently, maxillary central incisors are the teeth undergoing trauma because of their location in the arch, and hence are predisposed to developing ICR [9]. The exact nature of the resorptive lesion is still an area of debate, because this condition hasbeen termed as an inflammatory reaction [10], aseptic resorptive lesion [11], or a pathology stimulated by micro-organisms from the gingival sulcus, or the pulp space in the teeth with a necrotic pulp [3].

It has been suggested that the necrotic pulp has no role in stimulating or sustaining cervical resorption [12] and that the pre-existing necrotic pulp would lead to external cervical resorption instead of ICR [2]. 

Classically, in ICR, the pulp would remain vital, because of a layer of pre-dentin protecting the pulp [8] except in very large and invasive defects. An important aspect is that external cervical resorption would be accompanied by radiographically appreciable radiolucency in the affected tooth and the adjacent bone [2]. 

In this particular case, no clinical or radiological evidence of the invasive lesion penetrating into the pulp or affecting the adjacent bone could be appreciated.Since there is no previous dental record for this patient, it cannot be ascertained whether the pulpal necrosis secondary to trauma occurred before development of the cervical resorptive defect or both pathologies progressed simultaneously and independently. The fact that progress of the invasive defect seems to halt in the one-year follow-up radiography seems to indicate that the advancing granulation tissue could have been stimulated by not only the once damaged periodontal cells, but also the continuous leakage of noxious stimuli produced by the bacteria in the necrotic pulp into the periodontium through dentinal tubules, lateral and accessory canals. A similar situation arises during intra-coronal bleaching, where the bleaching agents penetrate the dentin from the inside and irritate the surrounding periodontium, resulting in cervical resorption [13]. 

The resorptive lesion was classified as being a class II ICR defect, since the resorption appeared to start just below the level of epithelial attachment and showed very little extension into the radicular dentine. As asserted by Heithersay, class I-class III resorptive defects have good outcomes. 

Since in this case, the resorptive defect was classified as class II, the prognosis was deemed favorable. [2]. The characteristic pinkish discoloration, which is usually attributed to an invasive cervical defect, was absent in our patient because the tooth was already discolored from the leakage of necrotic pulpal products into the surrounding coronal dentin [14]. As the degree of discoloration depends upon the length of time the pulp has remained necrotic, the incisors in our patient exhibited significant discoloration because of the trauma occurring early in life. Probing during clinical examination also did not reveal any pockets or sub-gingival defects; hence the diagnosis was made on the basis of peri-apical radiographs taken while utilizing the parallex technique. In such cases, where the clinical pathognomic signs of invasive cervical resorption are difficult to appreciate, as a result of super-imposition of some other pathology, the role of radiography for the correct diagnosis can be well appreciated. But the diagnostic ability of conventional radiography is limited as they provide a two-dimensional image of a three-dimensional object [15].CBCT, in such instances, would detect not only the presence of a resorptive defect, but also its extension in three dimension and whether or not it has invaded the pulp [16]. This information would prove vital in appropriate diagnosis and subsequent management [17]. Several approaches have been recommended for the management of ICR such as sub-gingival curettage, orthodontic extrusion to expose the defect, intentional re-implantation, application of 90% aqueous solution of trichloracetic acid and the use of calcium hydroxide to neutralize the toxic products and eliminate the microorganisms [18-20]. Trichloracetic acid is frequently utilized in the management of ICR, because of the many advantages that it offers [21]. 

After surgically exposing the lesion, curettage of the invasive resorptive tissue is performed. Trichloracetic is carefully applied topically to the exposed defect using a mini-applicator or a small cotton pellet. Trichloracetic acid causes coagulation necrosis of the invasive tissue, making it avascular, along with inactivation of any potentially resorptive cells, decreasing the chance of recurrence. It also helps to control hemorrhage. Care must be taken while using trichloracetic acid because of its caustic nature. Orthodontic extrusion helps provide better access to the lesion and also improves the gingival and bony architecture [4]. In the majority of these techniques, the soft tissue needs to be manipulated in order to gain access to the defect. Numerous materials such as GIC, RMGIC, Geristore and MTA, employed in various procedures like reverse sandwich technique have been advocated to fill the defect. Since our patient refused surgical intervention, the option to raise a flap and fill the defect with any of the above mentioned materials could not be utilized. A rather, a guarded approach was adopted in which only the necrotic pulp was removed and the canal filled with obturating material. Once the canal was adequately cleaned and calcium hydroxide was used as an intra-canal medicament, the noxious stimulating factors from the necrotic pulp, entering the periodontal tissues, were eliminated [22]. This would explain the cessation of the invasive resorptive lesion. Thus, this approach could be used in patients in whom an aggressive surgical approach is either contra-indicated or deemed to be un-successful [23]. At the three year post treatment follow-up, both teeth were asymptomatic with no periodontal pockets circumferentially. Peri-apical Radiograph film showed healing of the peri-apical lesions which was present at the beginning of the treatment procedure. In addition, the invasive cervical defect on the mesial aspect of the central incisor had improved radio-opacity when compared with previous radiographs. This could be the result of bone or bone-like tissue in-filling the defect. Until the last follow-up, no signs were detectable of this radio-opaque tissue progressing into ankylosis of the tooth, but this adverse out-come should also be considered in the future follow-up examinations.

## CONCLUSION

In patients whom surgical exploration and subsequent treatment of invasive cervical resorption is not an option, ortho-grade endodontic therapy followed by use of calcium hydroxide as intra-canal medicament could offer an alternative modality of treatment. Further observation and follow-up of this particular case and other similarly provided treatments would offer new in-sight into the previously under-emphasized probable cause of continued stimulation of invasive cervical resorption, along with novel methods to eliminate progress of this pathology.
